# A Recombinant RABV-G-Based Chemiluminescence Immunoassay for Quantitative Detection of Rabies Virus Antibodies

**DOI:** 10.3390/vaccines14090814

**Published:** 2026-09-16

**Authors:** Yan Zhang, Lei Wang, Yanan Han, Na Feng, Ye Feng, Weiyao Sun

**Affiliations:** 1 School of Public Health, Beihua University, Jilin 132013, China; 2 Jilin Tongchuang Investment Co., Ltd., Changchun 130616, China; 3 College of Basic Medicine, Beihua University, Jilin 132013, China; 4 Key Laboratory of Jilin Province for Zoonoses Prevention and Control, State Key Laboratory of Pathogen and Biosecurity, Academy of Military Medical Sciences, Changchun 130122, China; 5 State Key Laboratory of Pathogen and Biosecurity, Academy of Military Medical Sciences, Beijing 100071, China

**Keywords:** rapid test kit, rabies virus, antibody level, quantification, specificity

## Abstract

Background: Rabies is a nearly 100% fatal zoonosis, and a rabies virus-neutralizing antibody (RVNA) titre of ≥0.5 IU/mL is the recognized correlate of protection, making post-vaccination serological monitoring essential. However, the gold-standard methods (RFFIT and FAVN) are time-consuming, require live virus, and are unsuitable for large-scale surveillance, while current ELISA kits often show poor concordance with neutralization tests. Methods: In this study, we established a chemiluminescence immunoassay (CLIA) employing magnetic beads coated with recombinant RABV glycoprotein (short-chain) and an acridinium ester-labeled rabbit anti-dog IgG. Results: The assay exhibited a detection limit of 0.24 IU/mL. Its performance was validated with 250 canine anti-RABV serum samples against the FAVN. The CLIA and FAVN results were strongly correlated (r = 0.9636, *p* < 0.0001), with a Deming slope of 0.96 (95% confidence interval: 0.92–1.00). The assay achieved a diagnostic sensitivity of 94.4% and a specificity of 97.1%. It demonstrated high accuracy (relative bias within ±10%), good repeatability (intra- and inter-assay coefficients of variation < 8%), and acceptable specificity, showing no cross-reactivity with canine parvovirus, canine distemper virus, or canine parainfluenza virus antibodies. Conclusions: This CLIA kit eliminates the need for live virus, yields quantitative results within 30 min, and offers simple operation and high-throughput capacity, making it a reliable and practical tool for rabies immune monitoring and scientific prevention and control.

## 1. Introduction

Rabies is an acute zoonotic disease caused by the rabies virus (RABV) that is nearly always fatal after clinical onset [1]. The WHO estimates ~59,000 human deaths annually, over 95% in Africa and Asia, with India and Nigeria bearing a heavy burden [2,3]. In China, cases declined by 94% between 2007 and 2020 but rose to 170 in 2024—the first increase since 2007—and ~40 million dog bites per year keep rabies a major public health threat [4,5]. Because no effective treatment exists, mass dog vaccination and post-exposure prophylaxis are the cornerstones of control, making post-vaccination antibody monitoring essential [6,7].

A rabies virus-neutralizing antibody (RVNA) titre ≥0.5 IU/mL is the internationally accepted protective threshold, and dog vaccination coverage > 70% is required to interrupt transmission [8,9]. Gold-standard methods (RFFIT and FAVN) reliably quantify RVNA but require live virus, high-level biosafety containment facilities, cell culture, 4–7 days turnaround, and skilled operators, rendering them impractical for large-scale surveillance or use in primary veterinary and resource-limited settings [10,11]. ELISA-based kits provide simpler, high-throughput alternatives, yet commercial assays show poor concordance with neutralization tests, especially high false-positive rates near the 0.5 IU/mL cutoff, largely due to the use of inactivated whole virus or purified glycoprotein that causes non-specific binding or conformational changes [12]. Colloidal gold tests are rapid but only qualitative or semi-quantitative [13]. Thus, a simple, rapid, accurate, and safe quantitative assay remains urgently needed.

Owing to its high sensitivity, wide linear range, simple and rapid operation, and freedom from stray light interference [14], chemiluminescence immunoassay (CLIA) has been increasingly adopted in veterinary and clinical diagnostics, with successful applications in detecting antibodies against viruses such as SARS-CoV-2 [15], African swine fever virus [16], and hepatitis B virus [17]. RABV glycoprotein (RABV-G) is the main target of neutralizing antibodies; recombinant RABV-G expressed in insect cells maintains native conformational epitopes and avoids handling live virus [18]. Here, we developed a CLIA based on short-chain recombinant RABV-G coated on magnetic beads and an acridinium ester-labeled detection antibody to form a sandwich complex, with chemiluminescence measured after magnetic separation. The kit showed a detection limit of 0.24 IU/mL. When 250 canine sera were tested and compared with FAVN, the correlation was r = 0.9636 (*p* < 0.0001) with a slope of 0.96 (95% CI: 0.92–1.00), yielding a diagnostic sensitivity of 93.3% and an overall agreement of 94.4%.

The assay demonstrated high accuracy (relative bias within ±10%), excellent repeatability (intra- and inter-assay CV < 8%), and no cross-reactivity with canine parvovirus, distemper virus, or parainfluenza virus antibodies. This 30 min, live-virus-free, high-throughput CLIA provides an objective and convenient tool for quantitative post-vaccination antibody assessment, offering strong support for rabies prevention and control.

## 2. Materials and Methods

### 2.1. Construction of Recombinant Baculovirus Expressing RABV-G

The ectodomain-coding region of rabies virus glycoprotein (RABV-G) from the ERA/1993 strain (GenBank: AAA47204) was modified by replacing residues 73–79 and 117–125 with flexible Gly-Gly-Ser-Gly-Gly linkers to enhance solubility and secretion. A gp67 signal peptide was fused to the N-terminus and a 6 × His tag to the C-terminus. The codon-optimized synthetic gene was cloned into pFastBac1. The resulting plasmid, pFastBac1-gG, was transformed into DH10Bac E. coli for site-specific transposition into bacmid. White colonies were selected on LB agar containing 50 μg/mL kanamycin, 7 μg/mL gentamicin, 10 μg/mL tetracycline, 100 μg/mL X-gal, and 40 μg/mL IPTG after 48 h incubation at 37 °C. Recombinant bacmid DNA was extracted using a bacmid extraction kit and verified by PCR with M13 forward/reverse and gene-specific primers.

### 2.2. Expression and Purification of Recombinant RABV-G

Sf9 (ATCC CRL-1711) cells were transfected with the recombinant bacmid using Cellfectin II (Invitrogen, Carlsbad, CA, USA), and baculovirus was amplified to the P2 generation in suspension culture. Then, the supernatant was harvested, clarified, and loaded onto a HisTrap HP (Cytiva, Marlborough, MA, USA) column equilibrated with 4 mM NaH_2_PO_4_, 16 mM Na_2_HPO_4_, 250 mM NaCl, 10 mM imidazole (pH 7.2). After washing with 60 mM imidazole, the His-tagged protein was eluted with 250 mM imidazole. Expression and purity were verified by SDS-PAGE, and Western blot analysis using an anti-His monoclonal antibody (GTX628914, GeneTex, Irvine, CA, USA). The immunoreactivity of the purified RABV-G was confirmed by probing blots with pooled canine anti-rabies sera (Laboratory storage) and HRP-conjugated anti-dog IgG (M212120s, Abmart, Shanghai, China).

### 2.3. Preparation of Magnetic Bead-Coated RABV-G (R1)

Purified RABV-G (100 μg in PBS, 1 mg/mL) was buffer-exchanged using a 50 kDa Amicon Ultra centrifugal filter (MilliporeSigma, Darmstadt, Germany) and then reacted with Sulfo-NHS-LC-LC-Biotin at a 20-fold molar excess for 2 h at 25 °C with gentle vortexing every 10 min. Unreacted biotin was removed by five cycles of ultrafiltration with PBS. For magnetic bead conjugation, 25 μL of streptavidin-coated magnetic beads were washed five times with PBS containing 0.05% Tween-20 (PBST) and resuspended with the biotinylated RABV-G in 200 μL PBST. The mixture was incubated for 1 h at 25 °C with end-over-end rotation. Unreacted streptavidin sites were blocked by adding 50 μL of CE210 blocking buffer overnight at 4 °C. The beads were then washed five times with PBST and resuspended in 250 μL of MES storage buffer (0.1 M MES, pH 6.5, 0.5% BSA) to a final concentration of 10 mg/mL. The working solution (R1) was freshly prepared by diluting the stock to 0.1 mg/mL with MES dilution buffer (0.1 M MES, pH 6.5, 0.5% BSA, 0.05% Tween-20).

### 2.4. Preparation of Acridinium Ester-Labeled Antibody (R2)

Rabbit anti-dog IgG (100 μg in PBS at 1 mg/mL) was buffer-exchanged using a 50 kDa Amicon Ultra filter. NHS-acridinium ester dissolved in DMSO at 10 mM was diluted to 0.2 mM in PBS immediately before use. A 10 μL aliquot of this working solution was added to the antibody and incubated for 2 h at 25 °C in the dark with intermittent vortexing. Unconjugated label was removed by five cycles of ultrafiltration with PBS. The purified conjugate was diluted to 100 μg/mL in MES storage buffer (0.1 M MES, pH 6.5, 0.5% BSA) and stored at 4 °C protected from light. The working solution (R2) was prepared at a concentration of 1 μg/mL in MES dilution buffer before use.

### 2.5. Chemiluminescence Immunoassay Procedure

The assay was performed on a fully automated chemiluminescence analyzer (Kesimai Smart 500S, Chongqing Keysmile Biotechnology Co., Ltd., Chongqing, China). A 100 μL aliquot of R1 and 2.5 μL of serum sample or standard were mixed and incubated for 10 min. Then, 100 μL of R2 was added and incubated for another 10 min. Following magnetic separation and three washes, chemiluminescence substrate was added and the relative light units (RLU) were measured.

### 2.6. Standard Curve and Quantification

Eight canine anti-rabies sera with RVNA titres pre-assigned by FAVN were used as calibrators. Each calibrator was tested in duplicate, and the RLU values were fitted using a four-parameter logistic (4-PL) model. Unknown sample concentrations were automatically interpolated from the standard curve; samples exceeding the upper limit were retested after appropriate dilution.

### 2.7. Clinical Samples and FAVN Testing

Two hundred and fifty canine serum samples were collected from pet hospitals during routine veterinary diagnostic procedures. All serum samples used in this study were pre-tested to determine neutralizing antibody titres using the FAVN. The WOAH reference serum of dog origin was purchased from the Nancy Laboratory for Rabies and Wildlife, France.

### 2.8. Sensitivity and Accuracy

The limit of detection (LOD) was determined by repeatedly measuring the zero calibrator (serum from unvaccinated neonatal dogs collected at a pet hospital) 10 times. The mean ($\bar{x}$) and standard deviation (SD) of the relative light units (RLU) were calculated, and the RLU value corresponding to $\bar{x}$ + 3.3 SD was interpolated from the dose–response curve to obtain the LOD in IU/mL. Accuracy was evaluated by testing each sample three times with the chemiluminescence assay, using the FAVN-derived value as the target concentration (T). The relative bias (B) was calculated as

B (%) = [(Xi − T)/T] × 100.

Xi is the mean of the three replicate measurements and the acceptance criterion for relative bias was set at ±15%.

### 2.9. Intra- and Inter-Assay Precision

Intra-assay precision was evaluated by measuring two serum samples with different RVNA levels ten times each using a single reagent lot. Inter-assay precision was evaluated by measuring the same two samples ten times each using three independent reagent lots. For both precision assessments, the mean ($\bar{x}$) and SD were computed, and the coefficient of variation (CV) was calculated as CV (%) = (SD/$\bar{x}$) × 100.

### 2.10. Specificity and Method Comparison

Cross-reactivity was assessed using monospecific canine sera positive for antibodies against canine parvovirus, canine distemper virus, or canine parainfluenza virus. These sera were obtained from dogs immunized with a single respective viral antigen and were kindly provided by Dr. Na Feng (Changchun Veterinary Research Institute). Three independent serum samples from different individuals were used to assess specificity. Method comparison was performed using the 250 clinical canine serum samples. All samples were tested with both the present CLIA kit and the virus neutralization tests reference method. Agreement between the two methods was evaluated by Passing–Bablok regression analysis, and the correlation was assessed by calculating Spearman’s rank correlation coefficient.

### 2.11. Diagnostic Sensitivity and Specificity

To determine the optimal diagnostic cutoff for the CLIA kit, receiver operating characteristic (ROC) analysis was performed using MedCalc (version: 20.0.14) with the FAVN results as the reference standard. The area under the ROC curve (AUC) was calculated, and the optimal cutoff was defined as the value that maximized the Youden index (sensitivity + specificity − 1).

### 2.12. Statistical Analysis

Data were analyzed using GraphPad Prism 9.0 version and MedCalc (version: 20.0.14). Continuous variables are expressed as means ± standard deviation. The 95% confidence intervals for slopes and the limits of agreement in Bland–Altman plots were calculated. All tests were two-tailed, and a *p*-value < 0.05 was considered statistically significant.

## 3. Results

### 3.1. Recombinant RABV-G Is Efficiently Expressed, Secreted, and Recognized by Rabies-Specific Antibodies

**Figure 1:**
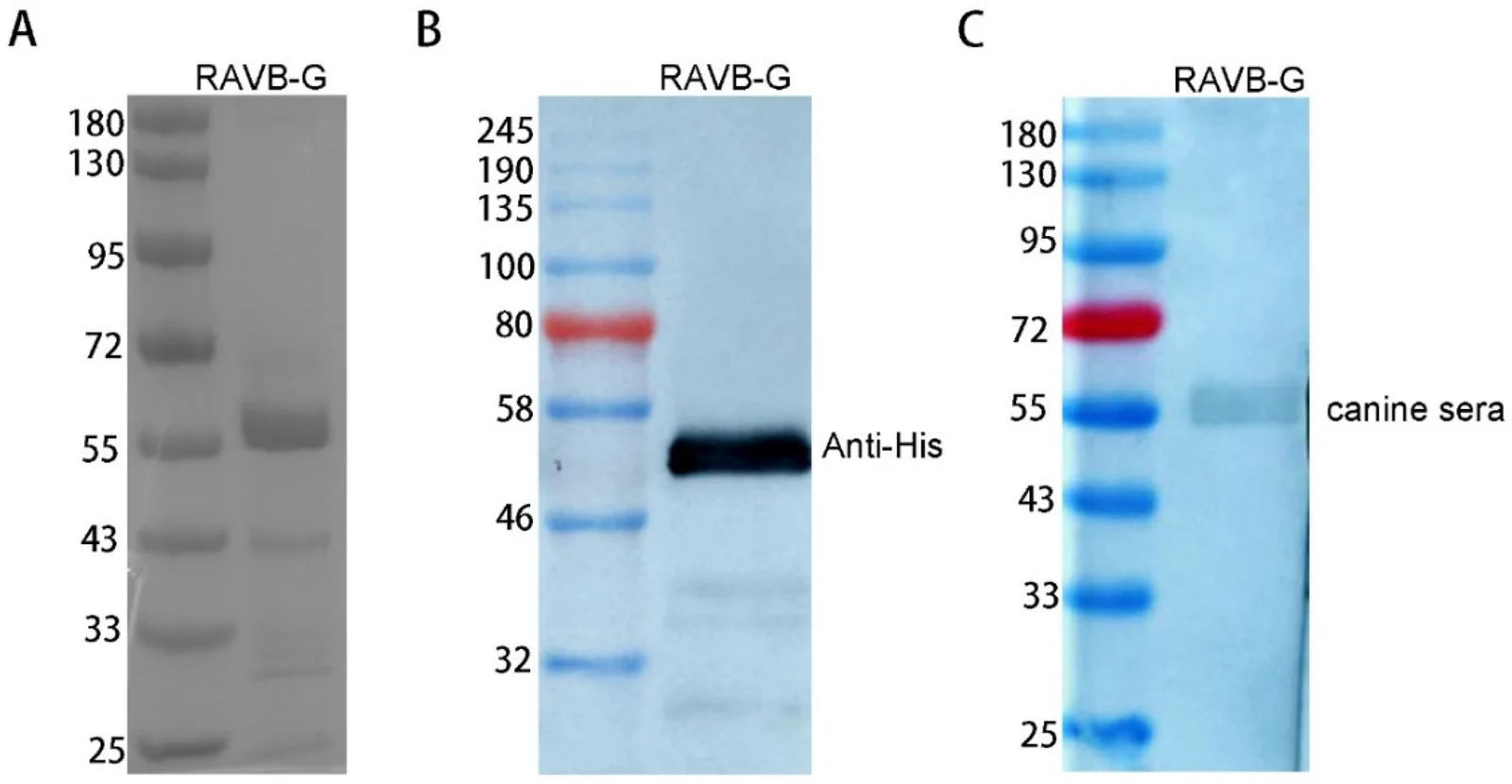
Purification and immunoreactivity of recombinant RABV-G. (**A**) SDS-PAGE analysis of purified RABV-G stained with Coomassie brilliant blue; WB analysis RABV-G using an anti-His monoclonal antibody (**B**); and canine anti-rabies positive sera (**C**).

The recombinant RABV-G was expressed in Sf9 cells using the baculovirus expression system and purified from the culture supernatant using Ni^2+^-affinity chromatography. SDS-PAGE analysis of the purified protein revealed a single band at approximately 55 kDa, corresponding to the expected molecular weight of the RABV-G ectodomain (Figure 1A). The identity of the purified protein was confirmed by WB using an anti-His monoclonal antibody, which detected a single specific band at the same position (Figure 1B). To further evaluate whether the recombinant protein retained antigenic reactivity, WB was performed using pooled canine anti-rabies positive sera as the primary antibody. A single distinct band at ~55 kDa was observed (Figure 1C), demonstrating that the purified recombinant RABV-G was specifically recognized by canine anti-rabies antibodies. Collectively, these results indicate that the recombinant RABV-G was successfully expressed and purified with high purity, and that it preserves the epitopes required for immunoreactivity with canine anti-rabies sera.

### 3.2. Establishment of a Four-Parameter Logistic Standard Curve Using FAVN-Calibrated Serum Standards

**Figure 2:**
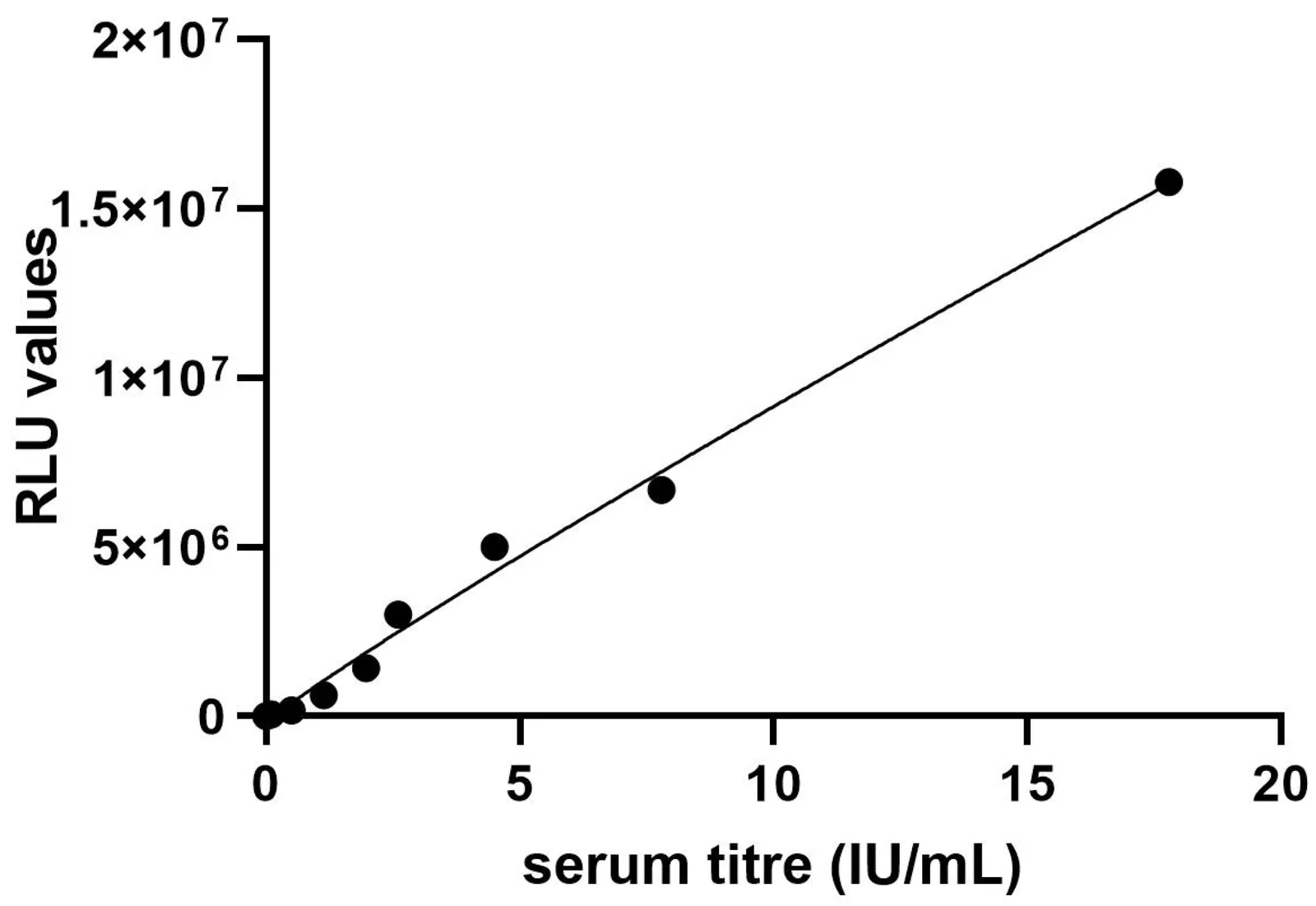
Four-parameter logistic (4-PL) standard curve generated with the serum titre. Relative light units (RLU) are plotted against the serum titre (IU/mL). The sigmoidal curve was fitted using a 4-PL regression model (top: 15952033818; bottom: −196417; EC50: 30851; HillSlope: 0.9262; R^2^ = 0.9924, degrees of freedom: 5). Each data point represents the mean of duplicate determinations.

To enable accurate quantification of rabies virus antibodies by the chemiluminescence immunoassay, a standard curve was constructed using seven canine anti-rabies positive serum control standards and a negative serum control standard. The assigned RVNA titres of these standards ranged from 0.1 to 17.79 IU/mL. Each standard was processed according to the CLIA procedure described above, and the chemiluminescent signal was recorded as relative light units. The dose–response relationship between the RLU values and the FAVN-assigned concentrations was fitted using a four-parameter logistic (4-PL) regression model in GraphPad Prism software. The resulting standard curve exhibited a characteristic linear scale with excellent goodness of fit (Figure 2), providing a reliable calibration function for converting RLU values of unknown samples into RVNA concentrations expressed in IU/mL.

### 3.3. The CLIA Kit Demonstrates a Low Limit of Detection and High Accuracy

To avoid biased performance estimates caused by the over-representation of high-titre samples, a panel of 250 canine serum specimens covering a wide range of RVNA concentrations was selected from our established serum bank and tested with the CLIA kit. All 250 sera had previously been assigned RVNA concentrations by the FAVN reference method. The limit of detection (LOD), defined as the concentration corresponding to the mean RLU of 10 zero-calibrator replicates plus 3.3 SD, was 0.24 IU/mL (Table 1). Accuracy was assessed using three sera with FAVN-determined target values of 0.5, 1.0 and 7.79 IU/mL. Three independent measurements gave relative biases of −5.08%, 6.20% and −4.71%, respectively (Table 2), both well within the predefined ±10% acceptance criterion, indicating excellent agreement between the CLIA kit and the FAVN reference method.

### 3.4. The CLIA Kit Exhibits Excellent Intra- and Inter-Assay Precision

Intra-assay precision was evaluated by testing two serum samples with different RVNA levels ten times each using a single reagent lot. Inter-assay precision was assessed by measuring the same two samples ten times each with three independently manufactured reagent lots. The CV was calculated for both assessments. As shown in Table 3, all CV values—both intra- and inter-assay—were below 5%, demonstrating that the CLIA kit provides highly reproducible results across replicate measurements and different production batches.

### 3.5. The CLIA Kit Shows No Cross-Reactivity with Antibodies Against Common Canine Viruses

The specificity of the CLIA kit was evaluated using monospecific canine sera positive for antibodies against canine parvovirus, canine distemper virus, and canine parainfluenza virus. For each virus, three independent serum samples from different individuals were tested (Table 4). The measured values for these non-target viral sera ranged from 0.33 to 0.39 IU/mL, remaining below the WHO clinical protective threshold of 0.5 IU/mL. These values were only marginally above the revised LOD of 0.24 IU/mL, a finding consistent with negligible background binding rather than meaningful cross-reactivity. Under the tested conditions, the kit demonstrated no clinically significant cross-reactivity with antibodies against these three common canine pathogens.

### 3.6. High Diagnostic Sensitivity and Specificity of the CLIA Kit Relative to FAVN

**Figure 3:**
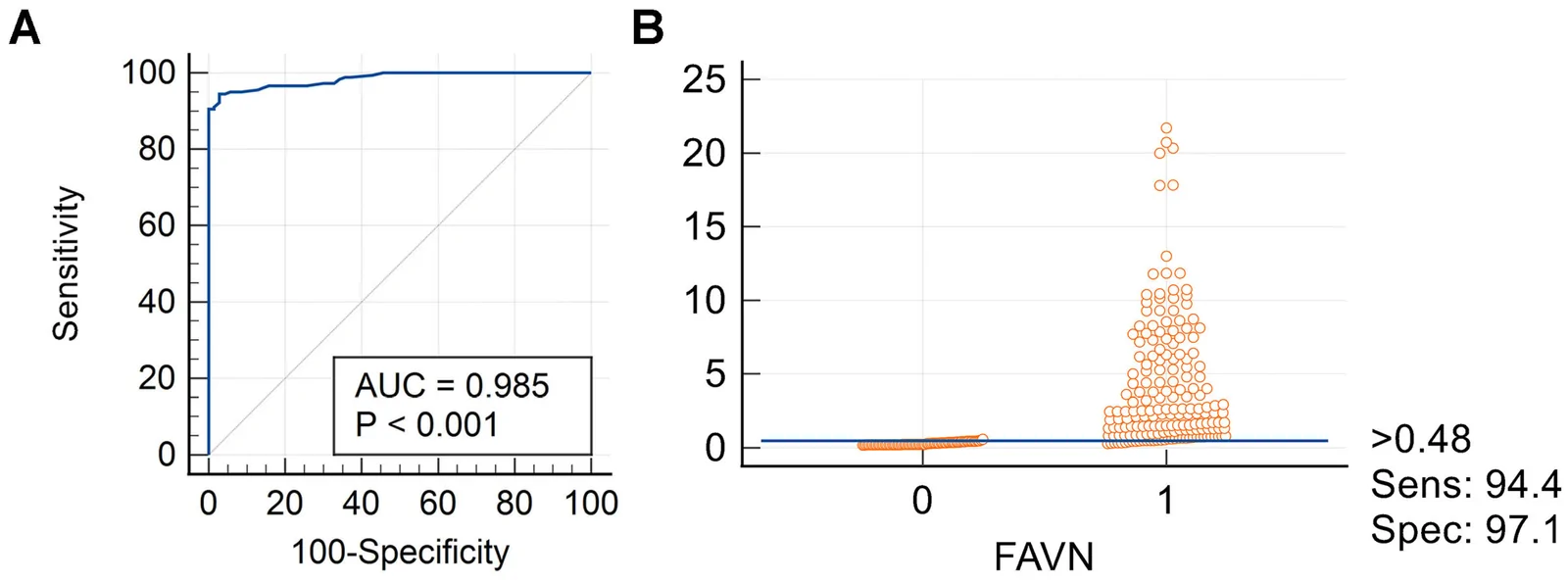
Diagnostic performance of the CLIA kit using FAVN as the reference method. (**A**) Receiver operating characteristic (ROC) curve (AUC: 0.985; standard error: 0.00563; Youden index J: 0.9159). (**B**) Interactive dot diagram showing the distribution of CLIA values for FAVN-negative and FAVN-positive samples. The blue solid line indicates the optimal cutoff (0.48 IU/mL) determined by maximizing the Youden index, corresponding to a sensitivity of 94.44% and a specificity of 97.14%.

To further validate the clinical applicability of the CLIA cutoff, receiver operating characteristic (ROC) analysis was performed using FAVN as the reference method. The ROC curve is shown in Figure 3A, and the corresponding interactive dot diagram is presented in Figure 3B. The area under the curve (AUC) was 0.985, indicating excellent discriminatory ability. The optimal cutoff determined by maximizing the Youden index was 0.48 IU/mL. Notably, using this cutoff, the 250 serum samples were classified as positive or negative, yielding 170 true positives, 10 false negatives, 2 false positives, and 68 true negatives. Accordingly, the diagnostic sensitivity was 94.4% and the specificity was 97.14%. These results demonstrate that the CLIA kit exhibits high diagnostic sensitivity and specificity relative to FAVN.

### 3.7. The CLIA Kit Demonstrates Strong Correlation and Agreement with the FAVN Reference Method

**Figure 4:**
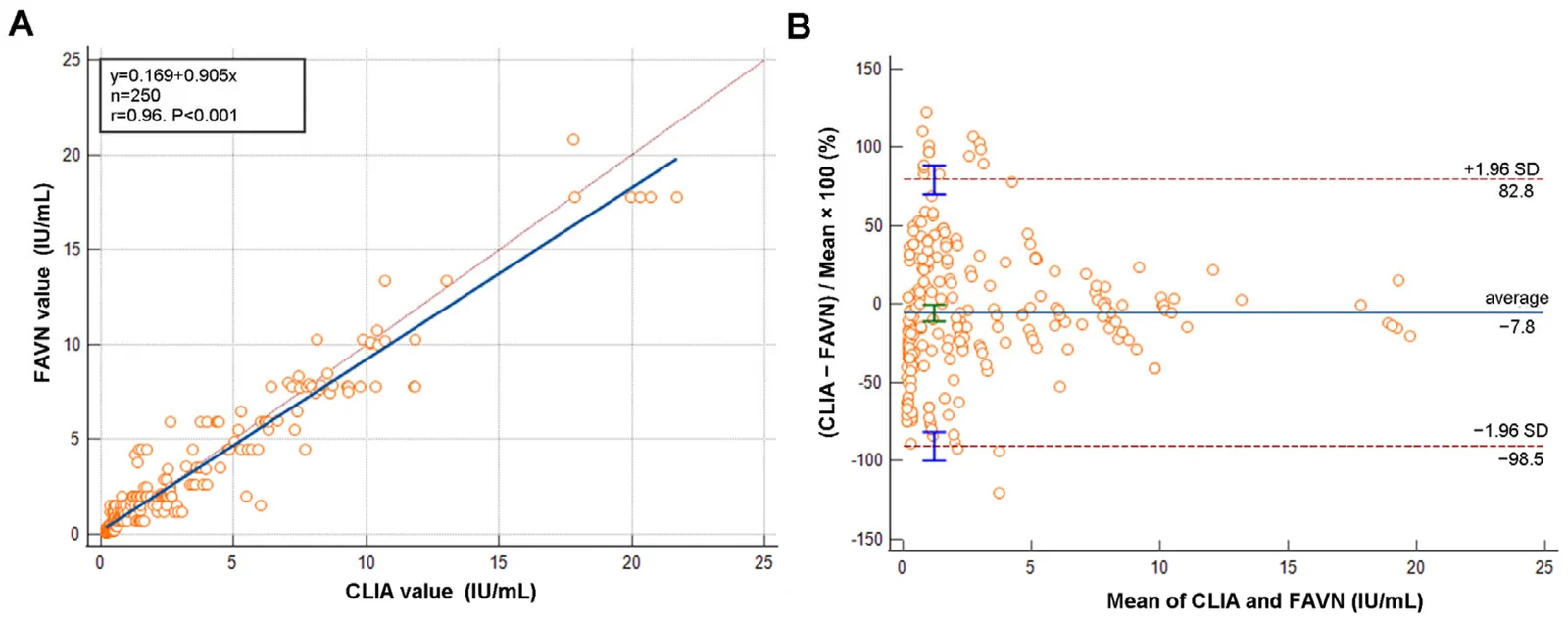
Method comparison between the CLIA kit and the FAVN reference method using 250 canine sera. (**A**) Scatter plot with Passing–Bablok regression; the blue solid line represents the Passing–Bablok regression fit and the brown dashed line represents the line of identity (y = x). (**B**) Bland–Altman difference plot.

The 250 canine serum samples were analyzed with the same lot of the CLIA kit, and the resulting RVNA concentrations were compared with the FAVN-assigned values (full dataset in Supplementary Table S1). Scatter plot analysis (Figure 4A) showed that the paired measurements covered the linear range of the assay without notable outliers, and the Bland–Altman difference plot (Figure 4B) indicated that fewer than 5% of the points fell outside the 95% limits of agreement. Spearman correlation analysis yielded a coefficient of r = 0.9636 (*p* < 0.0001). Passing–Bablok regression gave the equation y = 0.96x − 0.05, with a 95% confidence interval for the slope of 0.92–1.00 and for the intercept of −0.08 to −0.01. These results indicate excellent agreement between the CLIA kit and the FAVN method.

## 4. Discussion

Timely and accurate assessment of post-vaccination immunity is fundamental to rabies control, yet the widespread use of reference neutralization tests is hampered by the requirement for live virus, cumbersome procedures, and lengthy turnaround times [19]. In this study, we developed a fully automated chemiluminescence immunoassay (CLIA) based on a conformationally intact recombinant RABV-G and evaluated it against the FAVN gold standard using 250 canine sera. The main novelty of this work lies not only in introducing CLIA technology to rabies antibody detection, but more importantly in the rational design of the capture antigen—we expressed a soluble, secreted recombinant glycoprotein that preserves the critical neutralizing epitopes while completely eliminating the need for live virus. This strategy simultaneously addresses the biosafety risks inherent in traditional neutralization assays and overcomes the specificity limitations that have compromised many existing ELISA kits.

The central challenge in serological surveillance is reliably determining whether antibody levels meet the WHO-recommended protective threshold of 0.5 IU/mL [3]. Commercial ELISA kits, which commonly use inactivated whole virus or purified glycoprotein as coating antigens, tend to generate false-positive results near this cutoff owing to incomplete protein conformation and non-specific IgG binding [12]. In this work, we introduced flexible linker substitutions into the RABV-G ectodomain to improve solubility and immobilized the protein on magnetic beads via biotin–streptavidin oriented coupling, achieving a detection limit of 0.24 IU/mL—well below the protective threshold—and demonstrating no cross-reactivity with sera positive for canine parvovirus, distemper virus, or parainfluenza virus. Moreover, the chemiluminescence platform itself offers a wide linear dynamic range and a high signal-to-noise ratio, effectively narrowing the “gray zone” that often confounds test interpretation. These performance characteristics are particularly valuable for mass dog vaccination campaigns aiming at 70% coverage [9], as they enable animal health authorities to confidently identify inadequately protected individuals and promptly revaccinate them, thereby interrupting viral transmission within the dog population.

For method comparison, beyond calculating correlation coefficients, we performed Passing–Bablok regression and Bland–Altman analysis to assess proportional and systematic bias, respectively. The regression slope was 0.96 (95% CI 0.92–1.00) and the intercept was −0.05 (95% CI −0.08 to −0.01), indicating the absence of constant or proportional bias and confirming that the two methods are statistically interchangeable—a conclusion that cannot be drawn from correlation coefficients alone. The specificity reached 97.1% with a diagnostic sensitivity of 94.4%, comparable to or better than those of reported ELISA kits [20]; nevertheless, the 10 false-negative cases and the two false-positive cases merit further discussion. False-negative cases clustered near the threshold. Besides the differential detection of antibody isotypes, two additional mechanisms may contribute to these discordant results. First, sera containing low levels of potently neutralizing antibodies may produce strong neutralization in FAVN but relatively weak binding signals in CLIA, because CLIA reflects total binding antibodies rather than functional neutralization capacity. Second, some antibodies may preferentially recognize conformational epitopes present on the native virion surface but absent or poorly represented on the soluble recombinant RABV-G, leading to under-detection by CLIA. These possibilities could partly explain the outliers observed in Figure 4A. Similarly, the two false-positive samples also warrant clinical attention. A false-positive classification could incorrectly identify an animal with insufficient protective immunity as protected, thereby delaying or preventing timely revaccination. To minimize this risk, samples with values near the protective threshold or discordant results between CLIA and FAVN should be retested, and confirmatory neutralization testing is advisable.

Although the CLIA detects total binding IgG against RABV-G rather than functional neutralizing activity against the whole virion, it should not be regarded as a complete replacement for FAVN/RFFIT. Instead, it is best positioned as a practical screening tool for deciding canine revaccination schedules. In this proposed workflow, the high-throughput CLIA can be used to rapidly screen dogs after vaccination; those testing negative or near the 0.5 IU/mL cutoff can be immediately scheduled for booster vaccination, while those with equivocal or discordant results can be referred for confirmatory FAVN testing. This strategy combines the speed and scalability of CLIA with the functional specificity of the gold-standard assay, ensuring that animals with insufficient protective immunity are not missed while maximizing the efficiency of large-scale surveillance programs.

Beyond analytical performance, the operational advantages of this CLIA kit make it particularly suited for large-scale surveillance in rabies-endemic regions. In China alone, an estimated 40 million people are bitten by dogs each year [4,5], underscoring the urgent need for a deployable, decentralized testing tool. The assay requires only 2.5 μL of serum and delivers results on a fully automated platform within 30 min; critically, the entire procedure is live-virus-free, thereby eliminating the requirement for high-level biosafety containment facilities. It can therefore be implemented in primary veterinary clinics and field settings where RFFIT/FAVN are impractical, directly supporting routine herd immunity monitoring and evidence-based booster vaccination decisions. Future studies should validate the kit with sera from other susceptible species, evaluate its stability under field conditions, and explore its integration into comprehensive rabies surveillance systems.

## 5. Conclusions

In conclusion, we successfully developed a fully automated chemiluminescence immunoassay for the quantitative detection of anti-rabies virus antibodies, employing a soluble recombinant RABV-G antigen produced in insect cells. The assay demonstrated a low limit of detection (0.24 IU/mL), high accuracy (relative bias within ±10%), excellent intra- and inter-assay precision (CV < 5%), and no cross-reactivity against common canine pathogens. When applied to 250 canine sera, it exhibited a strong correlation with the FAVN reference method (r = 0.9636), a diagnostic sensitivity of 94.4%, and specificity of 97.1%. By eliminating the need for live virus and delivering results in 30 min in a high-throughput format, this CLIA kit provides a practical, safe, and reliable tool for large-scale post-vaccination antibody monitoring, holding great promise for supporting rabies surveillance and control programs.

## Figures and Tables

**Table 1:** RLU of 10 zero-calibrator replicates.

Zero Calibrator No.	RLU
1	81,900
2	74,359
3	84,867
4	75,242
5	74,921
6	83,103
7	85,108
8	73,076
9	76,124
10	85,108

**Table 2:** Accuracy of the CLIA kit evaluated with two canine anti-rabies sera.

T Value (IU/mL)	Xi (IU/mL)	Xi Average Value (IU/mL)	The Relative Bias B
0.50	0.48	0.47	−5.08%
	0.47		
	0.47		
1.00	1.09	1.06	6.20%
	1.06		
	1.04		
7.79	7.35	7.42	−4.71%
	7.39		
	7.53		

**Table 3:** Intra-assay and inter-assay precision of the CLIA kit.

Reference Positive Serums (IU/mL)	Intra-Assay			Inter-Assay		
	$\bar{x}$	SD	CV	$\bar{x}$	SD	CV
Sample 1	0.60	0.03	4.11%	0.60	0.02	3.78%
Sample 2	5.08	0.21	4.19%	5.12	0.24	4.77%

CV: coefficient variation; SD: standard deviation.

**Table 4:** Specificity of the CLIA kit against common canine pathogens.

Test Serum	Test Value IU/mL	Mean IU/mL
Canine parvovirus-positive serum	0.39	0.36
	0.36	
	0.33	
Canine distemper virus-positive serum	0.35	0.35
	0.35	
	0.34	
Canine parainfluenza virus-positive serum	0.34	0.35
	0.37	
	0.35	

## Data Availability

The original contributions presented in this study are included in the article. Further inquiries can be directed to the corresponding author.

## Supplementary Materials

The following supporting information can be downloaded at https://www.mdpi.com/article/10.3390/vaccines14090814/s1. Table S1. Individual RVNA concentrations of the 250 canine serum samples measured by the CLIA kit and the FAVN reference method.

## References

[1] Bisht K., Velthuis A.J.W.T. (2022). Decoding the Role of Temperature in RNA Virus Infections. mBio.

[2] World Health Organization (2013). WHO Expert Consultation on Rabies: Second Report.

[3] World Health Organization (2018). WHO Expert Consultation on Rabies: Third Report.

[4] Liu Z., Liu M., Tao X., Zhu W. (2021). Epidemic Characteristics of Human Rabies—China, 2016-2020. China CDC Wkly..

[5] Liu J.-J., Zhang N., Ding S.-J., Kou Z.-Q., Tao X.-Y., Zhu W.-Y. (2024). Epidemiological characteristics of human rabies cases reported by sites in China from 2006 to 2022. BMC Infect. Dis..

[6] Jackson A.C. (2018). Rabies: A medical perspective. Rev. Sci. Tech..

[7] Echevarría J.E., Banyard A.C., McElhinney L.M., Fooks A.R. (2019). Current Rabies Vaccines Do Not Confer Protective Immunity against Divergent Lyssaviruses Circulating in Europe. Viruses.

[8] Wasniewski M., Barrat J., Combes B., Guiot A.L., Cliquet F. (2014). Use of filter paper blood samples for rabies antibody detection in foxes and raccoon dogs. J. Virol. Methods.

[9] Al W. (2008). The rabies situation in Western Europe. Dev. Biol..

[10] Um J., Chun B.C., Lee Y.S., Hwang K.J., Yang D.-K., Park J.-S., Kim S.Y. (2017). Development and evaluation of an anti-rabies virus phosphoprotein-specific monoclonal antibody for detection of rabies neutralizing antibodies using RFFIT. PLoS Neglected Trop. Dis..

[11] Yang D.-K., Kim H.-H., Park Y.-R., Yoo J.Y., Park Y., Park J., Hyun B.-H. (2021). Generation of a recombinant rabies virus expressing green fluorescent protein for a virus neutralization antibody assay. J. Vet. Sci..

[12] Rodriguez M.C., Fontana D., Garay E., Prieto C. (2021). Detection and quantification of anti-rabies glycoprotein antibodies: Current state and perspectives. Appl. Microbiol. Biotechnol..

[13] Tao C., Li G. (2014). Rapid one-step immunochromatographic test strip for rabies detection using canine serum samples. Lett. Appl. Microbiol..

[14] Ye K., Shi D., Zhang Z., Bian L., Li Z., Liu T., He C., Xu S., Wu Y., Lin G. (2021). A chemiluminescence immunoassay for precise automatic quality control of glycoprotein in human rabies vaccine. Vaccine.

[15] Cristiano A., Pieri M., Sarubbi S., Pelagalli M., Calugi G., Tomassetti F., Bernardini S., Nuccetelli M. (2021). Evaluation of serological anti-SARS-CoV-2 chemiluminescent immunoassays correlated to live virus neutralization test, for the detection of anti-RBD antibodies as a relevant alternative in COVID-19 large-scale neutralizing activity monitoring. Clin. Immunol..

[16] Miao C., Shao J., Yang S., Wen S., Ma Y., Gao S., Chang H., Liu W. (2024). Development of plate-type and tubular chemiluminescence immunoassay against African swine fever virus p72. Appl. Microbiol. Biotechnol..

[17] Shen L., Zhang Y., Shi M., Shao L., Feng S., Li W., Fang Z., Yin J., Li T. (2024). Performance evaluation of the MAGLUMI Hepatitis B virus surface antigen chemiluminescence immunoassay. J. Med. Virol..

[18] Ng W.M., Fedosyuk S., English S., Augusto G., Berg A., Thorley L., Haselon A.-S., Segireddy R.R., Bowden T.A., Douglas A.D. (2022). Structure of trimeric pre-fusion rabies virus glycoprotein in complex with two protective antibodies. Cell Host Microbe.

[19] Su K., Xue J., Shan X., Ye H., Zhang L., Tan S., Shao J., Shi Y., Wang Z., Zhang L. (2021). Review of Detection and Quantification of Rabies Virus Antibodies. Viral Immunol..

[20] Xiao Y., Wu M., Xu W., Sun S., Liu T., Liu Y., Li H., Feng N., Tu C., Feng Y. (2025). Development and validation of a blocking ELISA for measurement of rabies virus neutralizing antibody. J. Clin. Microbiol..

