# Supplementary material for: A Recombinant RABV-G-Based Chemiluminescence Immunoassay for Quantitative Detection of Rabies Virus Antibodies
Source: Vaccines (Basel). 2026 Sep 16;14(9):814. doi: 10.3390/vaccines14090814 (PMC13612008; doi:10.3390/vaccines14090814)
Supplement: Supplementary file 1 [file vaccines-14-00814-s001.zip › vaccines-4507105-supplementary.pdf]

Supplementary Table S1. Individual RVNA concentrations of the 250 canine serum samples measured by the CLIA kit and the FAVN reference method

serum No.	FANV IU/mL	CLIA IU/mL	serum No.	FANV IU/mL	CLIA IU/mL	serum No.	FANV IU/mL	CLIA IU/mL	serum No.	FANV IU/mL	CLIA IU/mL
1	3.5	3.60	26	0.17	0.20	51	10.01	10.43	76	0.19	0.24
2	2.5	2.59	27	5.92	2.60	52	10.18	10.71	77	0.2	0.24
3	0.66	1.31	28	1.97	1.51	53	2.1	2.33	78	0.16	0.20
4	0.29	0.33	29	1.97	0.81	54	2.15	2.27	79	0.16	0.22
5	0.22	0.46	30	0.66	0.49	55	2.04	2.38	80	0.14	0.21
6	1.14	2.93	31	0.29	0.30	56	2.02	2.44	81	0.19	0.21
7	1.5	1.30	32	0.22	0.25	57	2.02	2.65	82	0.22	0.21
8	0.22	0.32	33	0.13	0.21	58	2.27	2.63	83	0.18	0.23
9	1.14	0.62	34	0.1	0.19	59	1.99	2.28	84	0.17	0.22
10	1.97	1.20	35	0.29	0.21	60	2.02	2.47	85	0.23	0.22
11	4.5	5.30	36	0.1	0.19	61	2.1	2.60	86	0.23	0.24
12	7.79	7.18	37	1.97	5.47	62	1.97	2.64	87	0.15	0.25
13	5.92	4.00	38	3.42	2.51	63	1.06	0.84	88	0.1	0.21
14	1.5	1.11	39	2.92	2.36	64	1.12	0.84	89	8.33	7.47
15	1.5	0.36	40	1.79	1.11	65	1.13	0.80	90	7.65	8.25
16	1.14	0.33	41	10.26	8.13	66	1.03	0.79	91	7.85	8.28
17	17.77	17.84	42	4.5	3.43	67	1.01	0.91	92	7.99	7.06
18	7.79	6.41	43	13.38	10.72	68	0.35	0.38	93	7.42	8.63
19	2.6	3.37	44	10.77	10.39	69	0.37	0.40	94	8.5	8.54
20	0.66	0.77	45	1.1	1.21	70	0.3	0.42	95	7.93	7.86
21	0.38	0.57	46	3.5	4.48	71	0.29	0.39	96	7.72	7.48
22	4.5	5.50	47	0.66	0.86	72	0.45	0.42	97	7.82	8.72
23	4.2	1.27	48	10.07	10.14	73	0.17	0.24	98	5.5	7.31
24	0.87	0.56	49	10.28	9.86	74	0.18	0.24	99	4.5	4.80
25	0.5	0.30	50	10.1	10.16	75	0.17	0.24	100	3.6	3.19

serum No.	FANV IU/mL	CLIA IU/mL	serum No.	FANV IU/mL	CLIA IU/mL	serum No.	FANV IU/mL	CLIA IU/mL	serum No.	FANV IU/mL	CLIA IU/mL
101	0.66	1.62	126	1.97	2.28	151	1.14	2.75	176	0.29	0.43
102	0.99	0.76	127	1.97	1.32	152	1.5	0.84	177	0.22	0.31
103	0.22	0.48	128	0.66	0.60	153	0.22	0.36	178	0.13	0.24
104	1.14	3.08	129	0.29	0.36	154	1.14	1.05	179	0.1	0.22
105	1.5	0.73	130	0.22	0.30	155	1.97	1.48	180	0.29	0.20
106	0.22	0.31	131	0.13	0.24	156	6.5	7.39	181	1.5	6.00
107	1.14	0.86	132	0.1	0.22	157	7.79	11.79	182	1.5	2.14
108	1.97	2.26	133	0.29	0.22	158	5.92	6.33	183	0.66	1.46
109	4.5	7.71	134	1.56	1.96	159	4.5	1.44	184	0.29	0.31
110	7.79	7.74	135	1.23	1.48	160	1.5	0.52	185	0.22	0.45
111	5.92	6.22	136	7.47	8.10	161	1.14	0.45	186	1.14	2.12
112	2.5	1.63	137	0.66	0.98	162	17.77	21.70	187	1.5	0.96
113	1.5	0.49	138	5.97	6.67	163	7.79	9.75	188	0.22	0.38
114	1.14	0.47	139	3.42	3.94	164	2.6	3.83	189	1.14	0.75
115	17.77	19.98	140	2.92	2.45	165	0.66	1.47	190	1.97	1.67
116	7.79	9.29	141	3.79	1.36	166	0.38	0.52	191	7.5	9.31
117	2.6	4.00	142	10.26	11.85	167	5.5	6.30	192	7.79	9.30
118	0.96	1.29	143	3.5	3.77	168	4.5	1.53	193	5.92	6.02
119	0.68	0.65	144	13.38	13.00	169	0.87	0.70	194	1.5	1.49
120	4.9	5.02	145	20.77	17.81	170	0.5	0.31	195	1.5	0.52
121	4.5	1.72	146	4.5	4.81	171	0.17	0.22	196	0.74	0.43
122	0.87	0.69	147	1.5	2.86	172	5.92	4.35	197	17.77	20.72
123	0.5	0.51	148	0.66	1.53	173	1.97	2.50	198	7.79	10.35
124	0.17	0.23	149	0.29	0.35	174	1.97	1.36	199	2.6	3.45
125	5.92	4.44	150	0.22	0.47	175	0.66	0.61	200	0.66	1.41

serum No.	FANV IU/mL	CLIA IU/mL	serum No.	FANV IU/mL	CLIA IU/mL	serum No.	FANV IU/mL	CLIA IU/mL	serum No.	FANV IU/mL	CLIA IU/mL
201	0.38	0.45	226	4.5	5.65						
202	6.5	5.28	227	7.79	7.94						
203	1.5	1.98	228	5.92	6.16						
204	0.87	0.75	229	1.5	1.44						
205	0.5	0.34	230	0.87	0.51						
206	0.17	0.22	231	1.14	0.44						
207	5.92	4.41	232	17.77	20.34						
208	1.97	2.14	233	7.79	11.83						
209	1.97	1.20	234	2.6	3.56						
210	0.66	0.66	235	0.66	1.54						
211	0.29	0.36	236	0.38	0.46						
212	0.22	0.29	237	5.5	5.20						
213	0.13	0.25	238	2.5	1.71						
214	0.1	0.21	239	0.87	0.65						
215	0.29	0.21	240	0.5	0.34						
216	4.5	5.94	241	0.17	0.44						
217	1.5	2.45	242	5.92	3.76						
218	0.66	1.31	243	1.97	1.89						
219	0.29	0.40	244	1.97	1.24						
220	0.22	0.45	245	0.66	0.76						
221	1.14	2.38	246	0.29	0.35						
222	1.5	0.82	247	0.22	0.31						
223	0.22	0.34	248	0.13	0.25						
224	1.14	0.76	249	0.1	0.20						
225	1.97	1.72	250	0.29	0.21						
